# Validation of the Double Mediation Model of Workplace Well-Being on the Subjective Well-Being of Technological Employees

**DOI:** 10.3389/fpsyg.2022.838723

**Published:** 2022-02-07

**Authors:** Shu-Ya Chang, Hsiang-Chen Hsu

**Affiliations:** Department of Industrial Management, I-Shou University, Kaohsiung, Taiwan

**Keywords:** workplace well-being, job involvement, flow, subjective well-being, technological employee

## Abstract

In recent years, workplace well-being has been a popular research topic, because it is helpful to promote employees’ welfare, thereby bringing valuable personal and organizational outcomes. With the development of technology, the technology industry plays an important role in Taiwan. Although the salary and benefits provided by the technology industry are better than other industries, the work often requires a lot of time and effort. It is worth paying attention to whether a happy workplace will bring subjective well-being for the technology industry in Taiwan. This study explored the influence of workplace well-being, job involvement, and flow on the subjective well-being. The research was conducted by a questionnaire survey. A total of 256 employees in the technology industry in the Nanzi Processing Zone in Kaohsiung City, Taiwan were surveyed. Collected data were analyzed by statistical methods, such as multivariate and structural equation models. The study results indicated that workplace well-being, flow, and job involvement have a positive and significant impact on the subjective well-being. In addition to having a direct impact on subjective well-being, flow is also a significant variable to mediate the impact of workplace well-being to subjective well-being. In addition, job involvement also affects subjective well-being through flow, which means that the state of selflessness at work is the most important factor affecting subjective well-being. Finally, based on the research findings, the researcher provided practical suggestions to the government and the technology industry.

## Introduction

The advance of new technology makes humans live better. However, the development of technology relied on the dedication of employees who worked in the high-tech industry. According to a survey conducted by the Human Resources Bank of Taiwan, there are nearly 6 million overwork employees worked under high pressure in three major industries, including finance, technology, and communications.

They usually worked overtime and which caused them to have health problems, such as cardiovascular disease or stroke because of the high pressure of the industry. However, people ignore the pressure of the technology industry because its salary and benefits are much higher than those of traditional industries. The technology industry still attracts many young job seekers in Taiwan. To achieve sustainable development, the organization should avoid the loss of human resources and pay attention to the employees’ perception of well-being.

Well-being is what everyone desires most, and it is also the goal that people have always wanted to pursue. Li and Yu (2014) believed that in the past, people thought that if they avoided suffering, they would feel happy. However, modern people are more progressive in pursuing well-being. Butt et al. (2020) believed that happier people feel that work is more important. Therefore, the study of subjective well-being is widely used in organizational-related research since subjective well-being can effectively make employees actively participate in work so that productivity and performance would be improved. Also, it is related to whether the company can maintain a competitive advantage in the future, subjective well-being has received considerable attention within the organization (Kuykendall et al., 2015), and for its effect on the workplace performance (Okun, 2022).

In recent years, newspapers, magazines, and news media have often mentioned the term “happy enterprise” in Taiwan. A happy enterprise would provide a happy workplace that can help promote the well-being of employees, thereby bringing valuable atmosphere to the organization (Wilks and Neto, 2013). Taiwan 1,111 Manpower Bank cooperated with major news media, such as China Times to jointly promote several “happy enterprises” selection activities to commend happy companies. From the winner list of 2011 to 2021, most of them belong to the technology industry (Huang and Yen, 2018). The technology industry is trying hard to promote a happy workplace. Because the nature of the work is in line with the trend of the times, employees in the technology industry must constantly improve to keep up with the pace of the times, and their work pressure is relatively higher than in other industries. Although the salary and benefits of the technology industry are better than those of other industries, it is worth exploring whether it will increase the subjective well-being of employees. Therefore, this study uses the subjective well-being of employees in the technology industry as the dependent variable, and workplace well-being as the independent variable, to explore the relationship between workplace well-being and subjective well-being.

Job involvement is the attitude of the worker, which will be reflected in the work, organization, colleagues, and situation. Work input would affect the behavior of the worker (Hwang and Han, 2015; Wang and Chang, 2016). Hsu et al. (2019) believed that job involvement is more effective in assessing employees’ work attitudes than job satisfaction. Then, Lin and Tien (2018) also pointed out that the work of all employees, with the company’s good leadership, would improve organizational performance. In the technology industry, if employees want to have outstanding work performance, they must also have work more. However, whether work input will bring more happiness remains to be further verified. This study uses job involvement as a mediating variable to explore whether the provision of a happy workplace will make employees more engaged in work and have a positive impact on subjective well-being.

Flow was defined as a source of energy that allows people to concentrate on what they want to do (Kim and Thapa, 2018; Wang et al., 2018). It can also be regarded as a pleasant state and experience. Kim and Hall (2019) studied the impact of consumer virtual reality (VR) travel hedonic behavior on continuous use and the impact of subjective well-being. It was found that the perceived pleasure of flow state has a significant impact on subjective well-being. It was also confirmed that if employees focus on the task’s flow experience, they will help improve their happiness at work (Kawalya et al., 2019). A meaningful feeling will bring intrinsic motivation and make employees work happily. To make them think that work is a pleasant experience, which is of great help to the improvement of happiness. Therefore, this research also explores the relationship between flow and subjective well-being, and whether the happy workplace will influence subjective well-being through flow. Based on the above, this research uses employees in the technology industry as the survey object to explore the dual-mediation model of the workplace well-being on subjective well-being, in order to understand the direct and indirect effects of workplace well-being on subjective well-being.

## Literature Review

### Subjective Well-Being

Subjective well-being is defined as individuals’ cognitive and affective evaluations of their own lives (Seligman, 2018). Subjective well-being refers to an individual’s subjective emotional perception of the overall life (Lin et al., 2014; Shang et al., 2018). It is everyone’s pursuit of life. Taheri et al. (2019) focused on the personnel of Iranian public organizations as the research object. The study found that both personal and organizational factors affect the subjective well-being of employees. Moreover, Horowitz (2016) explored the impact of five dimensions (individual task freedom, monetary compensation, work safety, low work intensity, and safe working conditions) on the subjective well-being of American employees’ work quality. The research results showed that the quality of work affected subjective well-being by improving social life, promoting physical health, and increasing leisure time. Then, Darvishmotevali and Ali (2020) explored the relationship between job insecurity, subjective well-being, and job performance with 250 hotel employees. The study found that subjective well-being has a positive impact on employees’ work performance. From the above literature discussion, subjective well-being would be affected by organizational factors or conditions, and subjective well-being would affect job performance. Also, Butt et al. (2020) believed that people who are more conscious of well-being would take their work more seriously, be more dedicated, and have higher job satisfaction. Therefore, the organization has turned its attention to how to ensure the well-being of employees has become an important task for management.

### Workplace Well-Being

Since employee well-being is highly correlated with job satisfaction (Tamariz et al., 2021), job performance, and productivity (Kuykendall et al., 2015), the company should provide an environment that can create happiness for employees to feel well-being in the workplace. Wok and Hashim (2015) stated that happiness in the workplace is also considered an essential element of successful organizations. A happy workplace can retain talent on the one hand and attract new employees on the other. Because employees’ expectations of the workplace are expanding, many are looking for jobs that offer personal development, fulfillment, and well-being (Slemp et al., 2015). Then, Li et al. (2020) studied 356 Chinese healthcare workers and found that non-workaholics’ perceptions of happiness in the workplace decreased as work intensity increased. Also, Singh et al. (2019) explored the relationship between self-efficacy and well-being in the workplace with 527 full-time executives in Indi. Research findings showed that self-efficacy has a significant relationship with workplace well-being. Furthermore, the result suggests that the relationship between self-efficacy and workplace well-being was stronger among executives with a high level of sustainability practices and vice versa. Most school graduates would choose tech-related industry as their first job because the technology industry has better pay, benefits, and working environment compared with other industries. Therefore, this study determined workplace well-being as the independent variable and explore its effect on subjective well-being through flow.

### Job Involvement

According to Chukwusa (2020), job involvement means that employees not only work to earn a living but also more importantly, derive natural satisfaction from the work itself. They not only work on time but also are willing to work overtime without complaining. Lee et al. (2020) believed that job involvement is a key factor contributing to personal growth, satisfaction, and workplace goal-directed behavior. Moreover, Rehman et al. (2020) examined different leadership behaviors, job involvement, and performance of 757 Chinese and Middle Eastern managers and found that leadership behaviors affect employees’ job involvement and performance. Then, Lee et al. (2020) conducted a survey of 360 employees in the travel industry. The research results found that job involvement positively influences well-being. In summary, job involvement affects work performance and plays an important role in personal well-being. In addition, job involvement is associate with work-family conflict and workers’ age (Lambert et al., 2020).

### Flow

Kawalya et al. (2019) defined flow as the overall feeling that employees feel when they are fully engaged. According to Zhou (2019) and Gao et al. (2015), flow is an experience that people feel when they are fully focused on their actions. When a person is in a state of flow, people become absorbed in the activity they are engaged in and lose their perception of the outside world. In recent years, the concept of flow experience has been widely applied in social commerce (Zhou, 2019), online games (Chen et al., 2018), or leisure domain (Tang et al., 2019), and there are also studies that apply the concept of flow to the topic of work (Kawalya et al., 2019). Therefore, this study attempts to incorporate flow in to the model to investigate the effect of flow on subjective well-being and whether workplace well-being can further affect subjective well-being through flow.

### Research Hypotheses

#### Related Works of Workplace Well-Being and Subjective Well-Being

Workplace well-being and subjective well-being are more and more popular in academic and business settings. For example, Kun and Gadanecz (2019) studied the relationship between workplace well-being and psychological capital. They proved that workplace well-being would impact psychological capital significantly. The findings of Sahai and Mahapatra (2020) also indicated that job involvement and job satisfaction play important roles in workplace well-being. Workplace well-being is considered essential for a successful business and would increase employee psychological functioning and psychological well-being, including hedonic (e.g., affective and satisfaction; DiPietro et al., 2020) and affective (e.g., engagement; Wardani et al., 2020; Koon and Ho, 2021). The provision of workplace well-being contributes to the happiness of employees. The technology industry is likely to increase the subjective well-being of employees because of the excellent benefits, working environment, and promotion path it offers. Therefore, based on the above literature, this study proposes the hypothesis H1 as:

*H1:* Workplace well-being has a positive and significant impact on employees’ subjective well-being.

#### Related Works of Workplace Well-Being and Subjective Well-Being

The more employees work, the higher employees’ awareness of work characteristics, the more employees perceive intrinsic value, and the higher employees’ subjective well-being would be. Lambert et al. (2018) explored the relationship between work stress, job involvement on well-being. They found that there is a positive effect of job involvement on well-being. Moreover, a study by Zhou et al. (2019) found that employees perceive themselves to be closely related to the organization when their sense of identification with the organization is higher. Employees who want to be praised by the organization would be more engaged in their work, which in turn would lead to a greater sense of well-being. Then, Lee et al. (2020) explored the relationship between job involvement and well-being among cruise ship employees during their travel experience. Their research findings showed that working under a pleasant travel experience not only has a positive impact on job involvement but also produces a high sense of well-being. Therefore, this study proposes the hypothesis H2 as:

*H2:* Job involvement has a positive and significant impact on subjective well-being.

#### Related Works of Flow and Subjective Well-Being

Flow has a positive relationship to subjective well-being. For example, Kim and Hall (2019) found that consumers’ perceived pleasure of flow states had a significant effect on subjective well-being. Since the flow is to enter the “no-self state,” in this state, one forgets about time, space, and even oneself. When one focuses only on what he/she is doing, one feels a unity of mind and body, happiness, and joy. Kim and Hall (2019) explored the relationship between flow and subjective well-being using virtual reality (VR) travel as a target scenario. The study results also confirmed the highly significant effect of consumers’ perceived pleasure and flow state on subjective well-being. Moreover, Kawalya et al. (2019) explored the relationship between flow experiences on psychological capital and well-being in the workplace with a sample of 800 nurses. The findings revealed a significant positive relationship between flow experiences on psychological capital in the workplace and well-being. According to the above literature, this study proposes the hypothesis H3 as:

*H3:* Flow has a positive and significant impact on subjective well-being.

#### Related Works of Workplace Well-Being and Job Involvement

Though workplace well-being is multidimensional (Na’imah et al., 2021), it is relevant to job involvement. Brunetto et al. (2012) surveyed 193 police officers in Australia to examine the impact of emotional intelligence on job involvement and workplace well-being. Research findings indicated that emotional intelligence affects job involvement and workplace well-being. Moreover, Huang et al. (2016) targeted human resource managers/professionals in manufacturing, financial, and service companies in Taiwan to determine the relevance of workplace well-being and job involvement. The technology industry often invests a lot of resources to promote work and life balance outside of work, which will make employees more willing to commit to their work. Therefore, this study proposes the hypothesis H4 as:

*H4:* Workplace well-being has a positive and significant impact on job involvement.

#### Related Works of Workplace Well-Being and Flow

There is a correlation between workplace well-being and mind flow. Wok and Hashim (2015) suggested that the focus of workplace well-being is on the individual’s experience of the work environment. The concept is therefore similar to flow, as the components of flow include clear goals, clear feedback, a sense of control, etc. All of these are consistent with the environment and conditions offered by workplace well-being. That is when an organization can provide enough conditions to make people feel well-being, then more employees have no worries in this environment. The more employees can show their best working ability, the more they can naturally experience joyful emotions at work, concentrate on work, have fun at work and forget about the passage of time.

Scholars pointed out that a happy and healthy workplace allows employees to develop themselves, interact interpersonally, and use skills. A good workplace should have reasonable goals, a balance of personal safety, supportive supervision, adequate rewards for work, and meaningful work development. When employees work in such a workplace, they should be able to devote themselves. Therefore, if employees in the technology industry could experience workplace well-being, they would spend most of their time at work. This study proposes the hypothesis H5 as:

*H5:* Workplace well-being has a positive and significant impact on flow.

#### Related Job Involvement and Flow

Job involvement is the degree to which a person is fully dedicated to his or her work. If employees gain great job satisfaction, they are willing to put more effort into the work or involve themselves more in the job with no complaints. Flow is the state of total concentration and forgetfulness when doing something. Flow is more than just work input; it is the overall feeling that employees have when they are doing their best. It can infer that the higher the job involvement, the more satisfied employees can receive, and which makes them experience more flow under such a workplace.

Therefore, there is a correlation between job involvement and the state of flow. Fan (2013) investigated the relationship between organizational belongingness, mobility experience, and positive behavior of salon employees in a Taiwanese salon organization. The research findings showed that flow experience is highly correlated with employee motivation and the ability to meet workplace challenges. Moreover, Peifer et al. (2020) studied how incomplete tasks at work were related to flow experiences with 93 employees as subjects. The results of the study showed that a person’s job involvement in an unfinished task was associated with flow experiences. Accordingly, it can be inferred that as employees in the technology industry become more engaged in their work, if they can enter the state of flow. Therefore, this study proposes the hypothesis H6 as:

*H6:* Job involvement has a positive and significant impact on flow.

**Figure 1:**
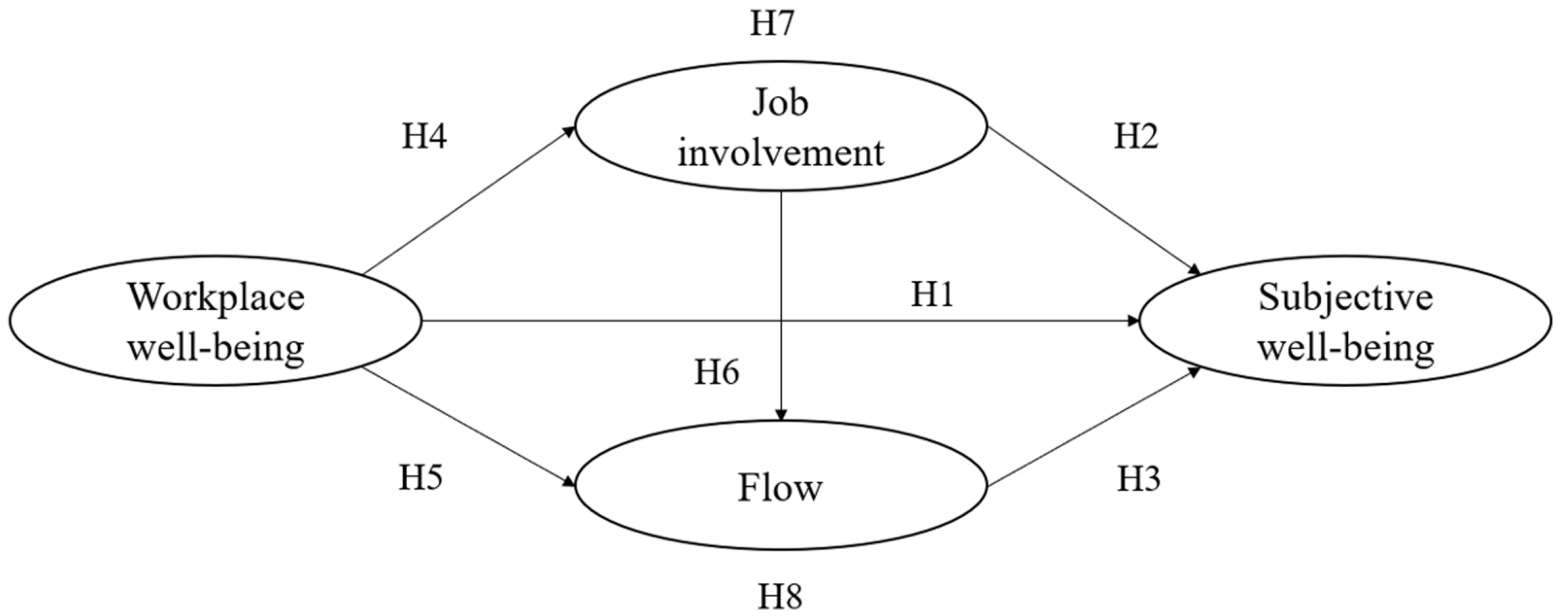
Research framework.

Table 1 shows the research hypothesis and its references, and Figure 1 displays the research framework.

**Table 1:** Summary of research hypothesis literature.

Hypothesis	Variable relationship	References
H1	Workplace well-being→Subjective well-being	Kun and Gadanecz (2019) and Sahai and Mahapatra (2020)
H2	Job involvement→Subjective well-being	Wang and Chang (2016), Zhou et al. (2019), and Lee et al. (2020)
H3	Flow→Subjective well-being	Kawalya et al. (2019) and Kim and Hall (2019)
H4	Workplace well-being→Job involvement	Brunetto et al. (2012), Huang et al. (2016), and Darvishmotevali and Ali (2020)
H5	Workplace well-being→Flow	Wilks and Neto (2013) and Wok and Hashim (2015)
H6	Job involvement→Flow	Fan (2013) and Peifer et al. (2020)

## Research Design

### Questionnaire Design

The questionnaire is divided into two parts: the first part is the basic personal information of technology employees in terms of gender, marriage, job title, and education level. The second part is about the factors impacting the subjective well-being of employees in the technology industry, which are three dimensions, namely, workplace well-being, job involvement, and flow. The questionnaire was designed using a seven-point Likert scale, with “1” indicating strongly disagree and “7” indicating strongly agree. After the design of the questionnaire was completed, it was reviewed by industry experts and scholars and gradually revised for improvement. The questionnaire contains four dimensions, 24 measurement indicators, and literature sources are shown in Table 2.

**Table 2:** Summary of measurement indicators.

Variable	No.	Indicator	References
Subjective well-being	SWB1	In most respects, my life has been close to ideal	Butt et al. (2020)
	SWB2	I think my living conditions are very good	
	SWB3	I am satisfied with my life	
	SWB4	So far, I’ve gotten the important things I want in life	
Workplace well-being	WWB1	My company’s salary package is better than the industry	Chiou (2014) and Huang and Yen (2018)
	WWB2	My company takes employee benefits very seriously	
	WWB3	My company provides education and training and cares about the growth of our employees.	
	WWB4	My company has a flexible working hours system that allows for more autonomy at work	
	WWB5	My company has an open line of communication that allows employees to fully express their opinions	
	WWB6	My company offers opportunities for promotion and development	
	WWB7	I get along well with my workmates	
	WWB8	My company would invest in social care and give back to society	
Job involvement	JIN 1	I like to focus on my work most of the time	Zopiatis et al. (2014)
	JIN 2	I think my work is very important to me	
	JIN3	Most of my personal goals in life are work-oriented	
	JIN 4	I have a very close connection with my current job	
	JIN 5	Most of my interests revolve around my work	
	JIN6	Work is everything to me	
Flow	FLOW1	I know exactly what I need to strengthen in my work	Chang (2017) and Lin et al. (2019)
	FLOW2	When working, I think time passes quickly	
	FLOW3	When working, I feel that time is not enough	
	FLOW4	When working, I enjoy it	
	FLOW5	Working in the field relaxes my mind and body	
	FLOW6	Working in the job can meet my wants	

### Sampling Method

This study focuses on the subjective well-being of employees in the technology industry; therefore, this study takes employees in the technology parks in southern Taiwan as the target population. The questionnaires were distributed in paper form from July 1 to July 31, 2021. During this period, the epidemic was controlled well in Taiwan, and the technological employees were affected to a very limited extent. The study adopted a cluster and convenience sampling method and was distributed with the assistance of the company’s human resources manager. A total of 300 questionnaires were distributed in total, excluding 44 questionnaires that were incompletely filled out and those with the same answer options. A total of 256 valid questionnaires were finally collected, with a valid questionnaire collection rate of 85%.

### Data Analysis

The data analysis methods used in this study included multivariate analysis (SPSS) and structural equation modelling (SEM). In the SPSS section, this study would conduct frequency distribution and item statistical analysis, and in the SEM section, measurement models (validated factor analysis) and structural models were included. The measurement model would consider the appropriateness of each measure for the interpretation of the components. The structural model can specify the relationship between the components.

## Results

### Descriptive Statistical Analysis

From the frequency distribution table, the basic information of the survey includes four items, which are gender, marital status, education level, and position. The largest number of respondents were female, with 138 (53.91%). The marital status, the largest number of respondents was married, with 132 (51.56%). Among the education level, the largest number of students, 152 (59.38%), was university students. Among the job position, the largest number of employees is at the basic level, with 170 (66.41%) as shown in Table 3.

**Table 3:** Frequency distribution table.

Variable	Value label	Value	Frequency	Valid percentage	Cummulated percentage
Gender	Male	1	118	46.09	46.09
	Female	2	138	53.91	100.00
		Total	256	100.0	
Marital status	Unmarried	1	124	48.44	48.44
	Married	2	132	51.56	100.00
		Total	256	100.0	
Education	Under high school	1	8	3.13	3.13
	Diploma	2	21	8.20	11.33
	University	3	152	59.38	70.70
	Master	4	68	26.56	97.27
	Doctor	5	7	2.73	100.00
		Total	256	100.0	
Job position	Basic employee	1	170	66.41	66.41
	Basic supervisor	2	47	18.36	84.77
	Middle supervisor	3	22	8.59	93.36
	Senior supervisor	4	17	6.64	100.00
		Total	256	100.0	

Table 4 below shows that the total number of valid questionnaires was 256. The mean value ranged from 4.02 to 5.22, the standard deviation ranged from 1.1 to 1.83, the skewness value ranged from −.72 to .48, and the kurtosis value ranged from −1.33 to −.21, which met the criteria of absolute skewness value less than two and absolute kurtosis value less than seven proposed by Kline (2015). This means that the data are consistent with the normal distribution. From the table below, the mean value of FLO02 is 5.22 and the mean value of JIN06 is 4.02, which means that the respondents agree most with FLO02 and less with JIN06. The lowest score is “Work is everything to me” and the highest score is “When working, I think time passes quickly.” This means that work is considered only a part of technological employees’ lives, and they are fully engaged in their works so they forget about the time.

**Table 4:** Descriptive statistics analysis.

Variable	*N*	Mean	Std. dev.	Kurtosis	Skewness
JIN01	256	5.11	1.32	−.94	−.17
JIN02	256	5.11	1.29	−1.02	−.22
JIN03	256	4.67	1.37	−.63	−.01
JIN04	256	4.80	1.27	−.84	−.01
JIN05	256	4.38	1.47	−.61	.04
JIN06	256	4.02	1.68	−.73	.02
WWB01	256	4.08	1.83	−1.08	−.07
WWB02	256	4.13	1.73	−.93	.08
WWB03	256	4.23	1.64	−.70	−.11
WWB04	256	4.15	1.67	−.78	−.01
WWB05	256	4.29	1.67	−.76	−.15
WWB06	256	4.11	1.68	−.87	−.14
WWB07	256	4.93	1.59	−.32	−.72
WWB08	256	4.38	1.58	−.59	−.26
FLOW01	256	5.21	1.10	−.46	−.04
FLOW02	256	5.22	1.19	−1.33	.19
FLOW03	256	4.95	1.15	−1.04	.40
FLOW04	256	4.59	1.33	−.41	.30
FLOW05	256	4.26	1.39	−.34	.34
FLOW06	256	4.53	1.31	−.44	.21
SWB01	256	4.76	1.10	−.29	.24
SWB02	256	4.63	1.22	−.21	.30
SWB03	256	4.71	1.15	−.41	.48
SWB04	256	4.64	1.35	−.39	.06

### Measurement Model

The most approximate estimation method was used to measure the model. The estimated parameters included factor loadings, reliability, convergent validity, and discriminant validity. According to the criteria proposed by Hair et al. (1998); Nunnally (1994) and Fornell and Larcker (1981) for convergent validity:

1. Standardized factor loading for each indicator variable should be greater than .50;
2. Composite reliability should be higher than .60; and
3. Average variance extracted should be higher than .50.

The standardized factor loadings of this study ranged from .604 to .947, all of which were within the range, indicating that each question had question reliability. The reliability of the study components ranged from .899 to .964, all of which exceeded .7 and met the criteria suggested by scholars, indicating that each component had good internal consistency. Finally, the average variance extractions ranged from .605 to .857, all of which were higher than .5 and met the criteria of Hair et al. (1998) and Fornell and Larcker (1981), indicating good convergent validity for each construct as shown in Table 5.

**Table 5:** Analysis results of the measurement model.

Construct	Item	Std. factor loading	SMC	CR	AVE
WWB	WWB01	.858	.736	.964	.772
	WWB02	.944	.891		
	WWB03	.947	.897		
	WWB04	.853	.728		
	WWB05	.926	.857		
	WWB06	.912	.832		
	WWB07	.707	.500		
	WWB08	.858	.736		
JIN	JIN01	.762	.581	.931	.695
	JIN02	.746	.557		
	JIN03	.902	.814		
	JIN04	.862	.743		
	JIN05	.881	.776		
	JIN06	.835	.697		
FLOW	FLOW01	.604	.365	.899	.605
	FLOW02	.646	.417		
	FLOW03	.606	.367		
	FLOW04	.898	.806		
	FLOW05	.898	.806		
	FLOW06	.930	.865		
SWB	SWB01	.917	.841	.960	.857
	SWB02	.924	.854		
	SWB03	.947	.897		
	SWB04	.914	.835		

Unstd, Unstandardized factor loadings; Std, Standardized factor loadings; SMC, Square Multiple Correlations; CR, Composite Reliability; and AVE, Average Variance Extracted.

In terms of discriminant validity, a more rigorous AVE method was used in this study. Fornell and Larcker (1981) stated that discriminant validity should also consider the correlation between convergent validity and construct. Therefore, it is recommended that the square root of AVE for each construct should be greater than the correlation coefficient between that construct and the other constructs. The root mean square of AVE for each construct of the diagonal of this study is larger than the correlation coefficient outside the diagonal, so each construct of this study has good discriminant validity as shown in Table 6.

**Table 6:** The discriminant validity of the measurement model.

	AVE	WWB	JIN	FLO	SWB
WWB	.772	**.879**			
JIN	.695	.789	**.834**		
FLO	.605	.703	.729	**.778**	
SWB	.857	.741	.720	.729	**.926**

The items on the diagonal in bold represent the square roots of the AVE; off-diagonal elements are the correlation estimates.

### Structural Model

This study applies the 194 international journal papers examined in Jackson, Gil-laspy, and Purc-Stephenson (Jackson et al., 2009) as the blueprint for applying the model fitness analysis and reports the results of this study using the nine most widely used fitness indicators. The model fit metrics should meet the recommended thresholds (Schumacker and Lomax, 2016), e.g., *χ*^2^ should be as low as possible, and since *χ*^2^ is very sensitive to large samples, it must be evaluated with chi-square values/degrees of freedom. Good model fitness chi-square value/degrees of freedom should be less than 3. After the Bollen-Stine Bootstrap modified model fit, all the fit indicators of this study were passed, indicating that the fit of this study was good as shown in Table 7.

**Table 7:** Model fit.

Model fit	Criteria	Model fit of the research model
ML *χ*^2^ Chi-square value	The small the better	389.280
DF Degrees of freedom	The large the better	246.000
Normed Chi-sqr (*χ*^2^/DF) Chi-square value/Degrees of freedom	1 < *χ*^2^/DF < 3	1.582
RMSEA	<.08	.048
SRMR	<.08	.080
TLI (NNFI)	>.9	.979
CFI	>.9	.981
GFI	>.9	.951
AGFI	>.9	.940

**Figure 2:**
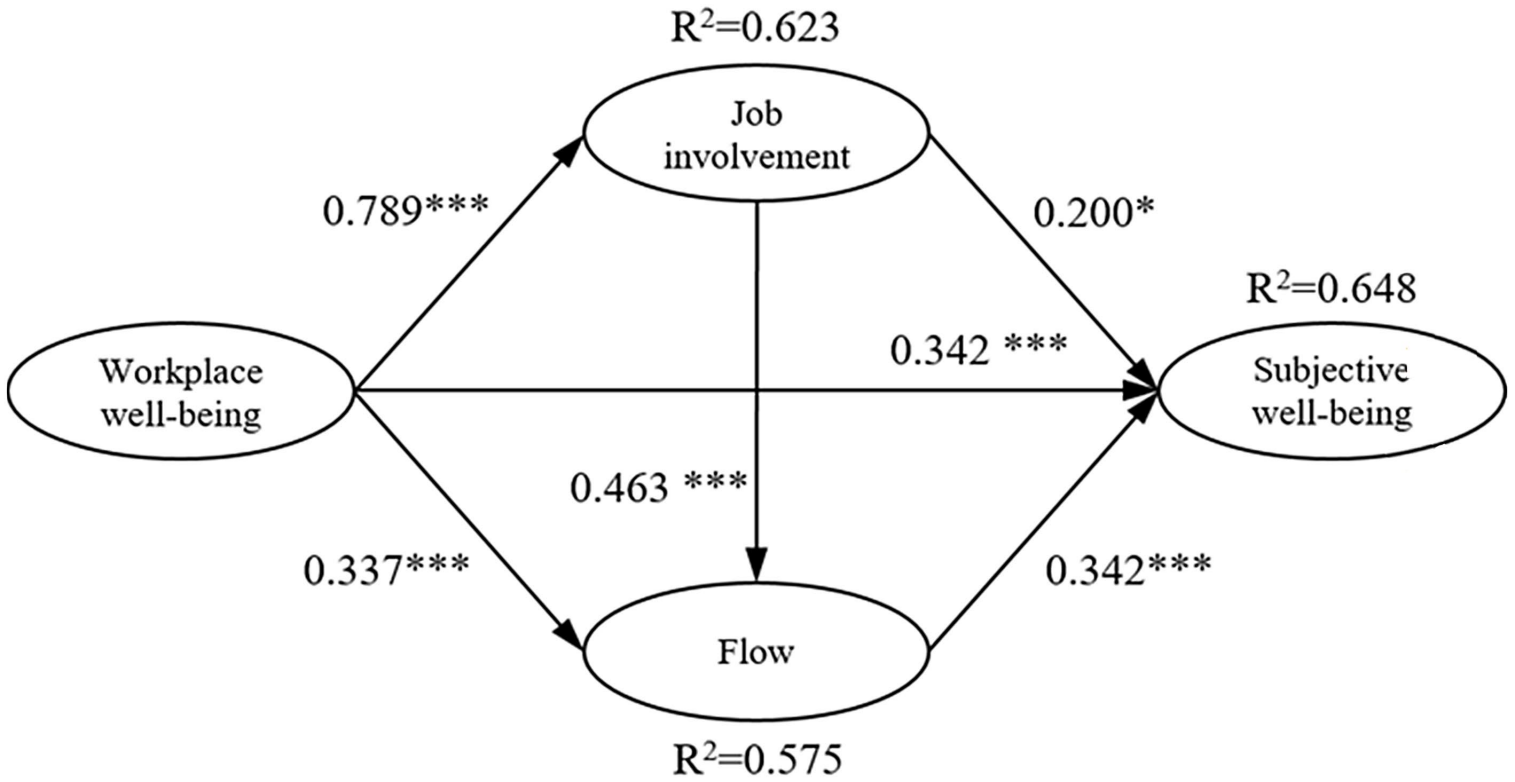
Research framework path analysis diagram.

The results of the path coefficient were calculated. Workplace well-being (*b* = .221, *p* < .001), job involvement (*b* = .202, *p* = .013), and flow (*b* = .522, *p* < .001) significantly influenced subjective well-being. Workplace well-being (*b* = .506, *p* < .001) significantly influenced job involvement. Workplace well-being (*b* = .143, *p* < .001) and job involvement (*b* = .306, *p* < .001) significantly affected flow. These results supported the research questions of this model. The explained variation of workplace well-being, job involvement, and flow to subjective well-being was 64.8%. The explained variation of workplace well-being to job involvement was 62.3%. The explained variation of workplace well-being and job involvement to flow was 57.5% as shown in Table 8. Research framework path analysis is shown in Figure 2.

**Table 8:** Path analysis.

Hypothesis	Path	Coefficient	Value of *p*	*R* ^2^	Result
H1	Workplace well-being→Subjective well-being	.342	.000	.648	Supported
H2	Job involvement→Subjective well-being	.200	.013		Supported
H3	Flow→Subjective well-being	.342	.000		Supported
H4	Workplace well-being→Job involvement	.789	.000	.623	Supported
H5	Workplace well-being→Flow	.337	.000	.575	Supported
H6	Job involvement→Flow	.463	.000		Supported

### Mediation Effect Analysis

In some indirect effect studies, bootstrapping has been shown to have more statistical power in determining indirect effects than causal and coefficient product methods (MacKinnon et al., 2004; Williams and MacKinnon, 2008). One of the greatest advantages of the self-help method is that the estimation of the indirect effects does not require a normative sampling allocation of the indirect effects unlike the coefficient product method (e.g., B–K method).

From the table below for the analysis of the indirect effect of the mediation model, in the total effect of WWB → FLO, its *p* < .05, this confidence interval does not contain 0 [.214–.384], which means that the total effect holds. In the total indirect effect of WWB → JIN → FLO, its *p* < .05 and this confidence interval did not contain 0 [.093–.241], indicating that the total indirect effect holds, representing that the mediating effect holds.

In the total effect of WWB → SWB, its *p* < .05, this confidence interval does not contain 0 (.411–.55), indicating that the total effect holds. For a particular indirect effect WWB → JIN → SWB, *p* > .05, the confidence interval contains 0 (−.006–.207), indicating that the indirect effect does not hold. WWB → FLO → SWB, *p* < .05, the confidence interval does not contain 0 (.033–.152), indicating that the indirect effect holds, representing that the mediating effect holds. WWB → JIN → FLO → SWB, *p* < .05, the confidence interval does not contain 0 (.041–.16), indicating that the indirect effect holds, representing that the mediating effect holds. In the total effect of JIN → SWB, its *p* < .05, this confidence interval does not contain 0 (.173–.587), which means that the total effect holds. In the total indirect effect of IN→SWB, its *p* < .05, this confidence interval does not contain 0 (.077–.301), which means the total indirect effect holds, represents the mediating effect holds as shown in Table 9.

**Table 9:** Indirect effect analysis.

Effect	Point estimate	Product of coefficients			Bootstrap 1,000 times	
					Bias-corrected 95%	
		SE	*Z*-value	Value of *p*	Lower bound	Upper bound
**Total effects**						
WWB → FLO	.298	.043	6.866	.000	.214	.384
**Total indirect effects**						
WWB → JIN → FLO	.155	.037	4.186	.000	.093	.241
**Direct effects**						
WWB → FLO	.143	.041	3.457	.001	.070	.243
**Total effects**						
WWB → SWB	.479	.035	13.821	.000	.411	.550
**Total indirect effects**						
WWB → JIN → FLO → SWB	.258	.052	4.988	.000	.160	.366
**Specified indirect effects**						
WWB → JIN → SWB	.102	.054	1.885	.059	−.006	.207
WWB → FLO → SWB	.075	.028	2.640	.008	.033	.152
WWB → JIN → FLO → SWB	.081	.028	2.846	.004	.041	.160
**Direct effects**						
WWB → SWB	.221	.061	3.628	.000	.117	.347
**Total effects**						
JIN → SWB	.361	.101	3.577	.000	.173	.587
**Total indirect effects**						
JIN → FLO → SWB	.160	.055	2.913	.004	.077	.301
**Direct effects**						
JIN → SWB	.202	.110	1.842	.065	−.014	.431

## Discussion and Conclusion

### Discussion

The main contribution of this study is to understand the factors of subjective well-being of employees in the technology industry. The main findings are as follows.

First, the employee’s workplace well-being has a positive and significant effect on their subjective well-being. The result is similar to the findings of Kun and Gadanecz (2019) and Sahai and Mahapatra’s (2020) study. This means that a good or bad work environment affects subjective well-being. Second, the employee’s job involvement has a positive and significant effect on their subjective well-being. This result is similar to the findings of Zhou et al. (2019), Wang and Chang (2016), and Lee et al. (2020), which represent that the more engaged one is in work, the higher the subjective well-being. Moreover, the employee’s flow has a positive and significant effect on their subjective well-being. The result is similar to those of Zhou et al. (2019), Wang and Chang (2016), and Lee et al.’s (2020) study. It represents the state of flow of a person is doing something, which affects subjective well-being.

Furthermore, employees’ workplace well-being has a positive and significant effect on job involvement. The research finding is similar to those of Brunetto et al. (2012), Huang et al. (2016), and Darvishmotevali and Ali’s (2020) research. It means that if the environment, benefits, and treatment in the workplace are very good, the employees’ life would be stable and make employees concentrate on their work. Then, an employee’s workplace well-being has a positive significant effect on flow. The result is similar to those of Wilks and Neto (2013) and Wok and Hashim’s (2015) study. The better the environment and conditions provided by the company, the better it is for the improvement of flow. Finally, employees’ job involvement has a positive and significant effect on their flow. This result is similar to the findings of Fan (2013) and Peifer et al.’s (2020) study. It means that when employees concentrate on the work, the employees would have a feeling of forgetfulness and forget that time is passing.

From the above results, the objective workplace well-being does affect the subjective well-being of employees in the technology industry. The commitment to work and the experience of flow are also important factors that affect subjective well-being. The employer could improve technological employees’ subjective well-being by creating a happy workplace, encouraging their work engagement, and enhancing their mind flow. In particular, a happy workplace has the greatest impact on work engagement, which means that a friendlier working environment in the technology industry can enhance employees’ work engagement. The results also illustrated the importance of a happy workplace, which echoes the reason why “happy companies” are elected annually as a benchmark for each business in Taiwan.

In addition, the results of the mediation analysis showed that the mediating effect played by flow was greater than the mediating effect of work engagement in the relationship between workplace well-being and subjective well-being. On the other hand, the present study also found that the mediating effect of flow plays a fully mediating role in job involvement and subjective well-being. The flow is the most important factor in the dual mediators of this study. The flow concentrates people’s attention to a focused point, so the external environment has very restricted effects on the individuals’ thoughts and perceptions. The flow enables employees to respond to only a specific goal and increase their happiness. Therefore, the flow is an important antecedent of subjective well-being. It is worthwhile for corporations to work on how to create a work situation that can make employees enter the flow. Therefore, if the workplace well-being provided by the technology industry can provide employees with a flow experience, the subjective well-being of employees can be greatly enhanced.

### Conclusion

Workplace well-being has a positive relationship with job involvement, flow, and subjective well-being. On behalf of the corporate world, it is important to start with workplace well-being to make employees more engaged in their work, to create a state of flow in their work, and to influence their subjective well-being. The results of this study confirm that Taiwan’s technology industry is on the right track to provide workplace well-being. For example, the company can create a good and happy working environment by offering employees better pay than their peers, allowing them to work flexible hours, or allowing the office environment to have a simple gym, a parent-child care center, a café, and afternoon tea time for employees to relax appropriately. Furthermore, the flow experience has a significant positive effect on subjective well-being. Employees need to work in a relaxed environment to improve their flow. Therefore, it is recommended that supervisors can delegate appropriate authority to allow employees to set goals and give them a high degree of autonomy, etc. This is because once employees can have the experience of flow, they can concentrate fully on their work and bring happiness. Finally, job involvement also has a significant positive effect on subjective well-being. So, it is recommended that companies can enhance employees’ interest in their work. The HR department can provide aptitude tests with credibility to place employees in the right position for the right job, with the right talent for the right job, and with timely rotations to help enhance the freshness of the job, which in turn increases the enjoyment of the job and enhances their job involvement.

## Research Limitations and Future Research Recommendations

Although the design and implementation of this study were rigorous, there are still some limitations and shortcomings that cannot be avoided. In terms of research data and sample characteristics, the results of this study were only be inferred to the southern part of Taiwan, and the sample characteristics may not represent the whole country. It is suggested that future research could sample technology employees from the other area or countries to explore the antecedent variables that affect the subjective well-being, so the related research regarding subjective well-being could be enriched.

## Data Availability Statement

The raw data supporting the conclusions of this article will be made available by the authors, without undue reservation.

## Ethics Statement

Ethical review and approval were not required for the study on human participants in accordance with the local legislation and institutional requirements. Written informed consent for participation was not required for this study in accordance with the national legislation and the institutional requirements.

## Author Contributions

S-YC: conceptualization, methodology, validation, writing—original draft preparation, writing—review and editing, and supervision. H-CH: investigation, data curation, formal analysis, and writing—review and editing. All authors contributed to the article and approved the submitted version.

## Conflict of Interest

The authors declare that the research was conducted in the absence of any commercial or financial relationships that could be construed as a potential conflict of interest.

## Publisher’s Note

All claims expressed in this article are solely those of the authors and do not necessarily represent those of their affiliated organizations, or those of the publisher, the editors and the reviewers. Any product that may be evaluated in this article, or claim that may be made by its manufacturer, is not guaranteed or endorsed by the publisher.
